# Impact of pharmaceutical policy interventions on utilization of antipsychotic medicines in Finland and Portugal in times of economic recession: interrupted time series analyses

**DOI:** 10.1186/1475-9276-13-53

**Published:** 2014-07-25

**Authors:** Christine Leopold, Fang Zhang, Aukje K Mantel-Teeuwisse, Sabine Vogler, Silvia Valkova, Dennis Ross-Degnan, Anita K Wagner

**Affiliations:** 1 Department of Health Economics, Gesundheit Österreich GmbH/Austrian Health Institute/WHO Collaborating Centre for Pricing and Reimbursement Policies, Vienna, Austria; 2 Department of Population Medicine, Harvard Medical School and Harvard Pilgrim Health Care Institute, Boston, USA; 3 Utrecht Institute for Pharmaceutical Sciences, WHO Collaborating Centre for Pharmaceutical Policy and Regulation, Utrecht, Netherlands; 4 IMS Institute for Healthcare Informatics, Pennsylvania, USA

**Keywords:** Finland, Portugal, Antipsychotic medicines, Interrupted time series, Utilization of medicines

## Abstract

**Objectives:**

To analyze the impacts of pharmaceutical sector policies implemented to contain country spending during the economic recession – a reference price system in Finland and a mix of policies including changes in reimbursement rates, a generic promotion campaign and discounts granted to the public payer in Portugal – on utilization of, as a proxy for access to, antipsychotic medicines.

**Methodology:**

We obtained monthly IMS Health sales data in standard units of antipsychotic medicines in Portugal and Finland for the period January 2007 to December 2011. We used an interrupted time series design to estimate changes in overall use and generic market shares by comparing pre-policy and post-policy levels and trends.

**Results:**

Both countries’ policy approaches were associated with slight, likely unintended, decreases in overall use of antipsychotic medicines and with increases in generic market shares of major antipsychotic products. In Finland, quetiapine and risperidone generic market shares increased substantially (estimates one year post-policy compared to before, quetiapine: 6.80% [3.92%, 9.68%]; risperidone: 11.13% [6.79%, 15.48%]. The policy interventions in Portugal resulted in a substantially increased generic market share for amisulpride (estimate one year post-policy compared to before: 22.95% [21.01%, 24.90%]; generic risperidone already dominated the market prior to the policy interventions.

**Conclusions:**

Different policy approaches to contain pharmaceutical expenditures in times of the economic recession in Finland and Portugal had intended – increased use of generics – and likely unintended – slightly decreased overall sales, possibly consistent with decreased access to needed medicines – impacts. These findings highlight the importance of monitoring and evaluating the effects of pharmaceutical policy interventions on use of medicines and health outcomes.

## Background

The current economic recession, which started in 2009, has led many European countries – especially the Southern and Mediterranean countries – to introduce austerity measures primarily in public sectors such as publicly funded health care systems. In the pharmaceutical sector, European social security systems face the difficult task of guaranteeing their citizens equitable and sustainable access to needed medicines while containing ever-rising pharmaceutical sector expenditures [1-4]. Ideally, pharmaceutical sector cost-containment policies would result in increased efficiency of spending, without limiting access to needed medicines [5].

European systems have chosen different strategies to contain medicines expenditures [6-9]. Changes in patient co-payments for and increases in value-added taxes (VAT) on medicines were among the most frequently implemented cost-containment measures in 2010 [10]. These measures tend to shift cost-burden to those who need medicines. Pharmaceutical sector cost-containment policies may thus achieve financial savings for the public health system, and may also have unintended effects in the form of decreased utilization of needed medicines when patients cannot afford to pay a higher share of medicines costs [11-16].

Antipsychotic medications are essential for treatment of severe chronic mental illness, such as schizophrenia, bipolar disorder and autistic disorders, which are among the leading major chronic diseases in Europe [17,18]. In addition, off-label use of antipsychotic medicines is common for posttraumatic stress disorder, anxiety, attention deficit hyperactivity disorder and mood disorders. Due to their high cost, antipsychotic medications represent a large component of public spending on medicines and are therefore a frequent target of cost-containment policies. Reimbursement restrictions may, however, force patients to forego treatment in light of increased out-of-pocket payments [19] or to shift to other possibly less appropriate or more costly treatments [20]. For example, Soumerai et al. found that a policy that required prior approval for reimbursement of specific atypical antipsychotics to control expenditures in one US state public insurance program resulted in increased rates of discontinuity of antipsychotic drug treatment [21]. These results are consistent with an earlier study of the effects of other policies on discontinuation of antipsychotic agents among patients with severe mental illness [22].

Our intent was to assess the impacts of different cost-containment policies implemented during the recession in Finland and Portugal on antipsychotic utilization within each country and to compare the magnitude of effects. The recent recession affected both countries differently. Finland did not experience major declines in gross domestic product (GDP) during the recession. Portugal, however, suffered a 2.9% decline in GDP growth between 2008 and 2009 and another decline of 1.7% between 2010 and 2011 [23] leading to a strict three year public budget savings plan between the Portuguese Ministry of Finance and the Troika (consisting of representatives of the European Commission, the European Central Bank and the International Monetary Fund) [24]. We focus on antipsychotic medicines, which are included in the public reimbursement systems in both countries, because of the public health relevance of antipsychotic disorders and their treatment.

On April 1, 2009, Finland implemented a reference price system whereby all medicines with therapeutic alternatives on the National Social Security’s reimbursement list were clustered according to therapeutic similarity based on each medicine’s indication and pharmacology. Medicines in clusters were considered substitutable. For each substitution group, a reference price was set at the price of the least expensive medicine in the cluster, with patients having to pay the difference for higher cost medications out-of-pocket. Danzon et al. highlighted that a hoped-for effect of reference pricing is that manufacturers, anticipating shifts to less expensive (mostly generic) products, decrease prices to not lose customers [25]. According to Pohjolainen et al., the average price level for all medications decreased significantly in the first four quarters after the implementation of the reference price system in Finland, which led to 109 million Euro savings [26,27]. In addition to establishing reference pricing, the National Social Security Scheme delisted the antipsychotic brand product Seroquel (quetiapine) from its reimbursement scheme on January 1, 2009, because the manufacturer did not decrease its price to that of the 40% less expensive generics when they became available [28,29].

In contrast, Portugal introduced several contemporaneous cost-containment policies: on October 15, 2010, the Portuguese National Authority for Medicines and Health Products (INFARMED) harmonized the reimbursement rates for antipsychotic medicines to 90% of charges [30,31]. Prior to the change, antipsychotic medicines were reimbursed at 37%, but in reality all patients received antipsychotic medicines for free as physicians could state certain pathologies (as listed in the legislation) on the prescription for which antipsychotic medicines were dispensed to the patient without co-payment. Following the change in reimbursement rates, no indication-specific co-payment exemptions were allowed. In addition, from September 15 to October 8 2010, INFARMED launched a television and radio campaign to promote generics (“you save, we all save”), informing the public about the preferred use of generics due to lower prices of generics as compared to originals [32]. Finally, on October 15, 2010 a 6% deduction of the maximum retail price took effect for medicines that had not already lowered prices earlier; this deduction did not affect the final consumer price and is a statutory discount granted by industry and supply chain actors to the public payer [33]. In the beginning of 2013 this deduction was still applied.

We hypothesized that the implementation of the reference price system and delisting of the brand product Seroquel in Finland, targeting product prices, would lead to an increase in the proportion of sales (by volume) of generics, but likely no reduction in overall utilization, that is, likely not pose barriers to antipsychotic medicines access. We hypothesized that the change in reimbursement rates in Portugal would lead to an unintended decrease in sales by volume of antipsychotic medications, because patients incurred higher co-payments after the policy changes, which would constitute a barrier to access for some. Finally, we expected an increase in the generic market share in Portugal as a result of the generic campaign.

## Method

### Data source

We analyzed monthly pharmaceutical sales in Finland and Portugal between January 2007 and December 2011 provided by IMS Health [34]. Data are generated through audits of aggregated purchases of registered medicines by retail pharmacies from wholesalers in each country. IMS audits cover 812 pharmacies or 100% of the retail market in Finland and 2,910 pharmacies or 99% of the retail market in Portugal. IMS MIDAS combines national audits into a globally consistent view of pharmaceutical markets [35]. IMS Health collects data on original products, generics identified by a brand name, generics identified by INN name with company name as a prefix or suffix and INN unbranded generics. Documentation on the IMS data collection and validation process is available upon request from the authors. Antipsychotic medicines were those in Anatomical Therapeutic Chemical (ATC) category N5A of the European Pharmaceutical Research Association (EphMRA) [36]. A detailed list of all products included in the study is attached in Additional file 1.

We extracted information on policy changes in each country from the WHO Collaborating Centre for Pharmaceutical Pricing and Reimbursement Policies (PPRI) and the IMS PharmaQuery databases. In addition, information on policy changes was verified by national experts from Finland and Portugal through written communication.

### Outcome measures

We used two outcome measures for access: total volume of antipsychotic medicines per capita and percentage market share by volume in a therapeutic class. For the total volume analyses, we divided the total number of standard units (SU) sold per month by size of the national population to control for population growth, using annual populations estimated from the Organization for Economic Co-operation and Development (OECD) [36]. Our analyses included prescription-only antipsychotic medicines limited to the retail market, which represented 98% of antipsychotic medicines sales by volume in Portugal and 87% in Finland in 2011. A standard unit, as defined by IMS Health, is the smallest dose of a product, equivalent to one tablet or capsule for an oral dosage form, one teaspoon (i.e. 5 ml) for a syrup and one ampoule or vial for an injectable product. For the purpose of this study we grouped all types of generics (branded and unbranded) under the group “generics” and contrasted them with originator brand product use. We did so since only few unbranded generics were on the countries’ markets and followed the same trends as branded generics. We did no separate analyses of first versus second generation antipsychotic (SGA) medicines since the majority of products sold and the top selling products in each country were SGAs. Percent market share is the percent of total volume in the retail market for each active substance in two categories – originator brand products, which can be protected or no-longer-protected by patents depending on their exclusivity status in each period and country, and generic products which are not subject to patent protection.

### Study design and data analysis

We used an interrupted time series design, the strongest quasi-experimental design [37], to estimate changes in sales attributable to the policies by comparing sales after the interventions to estimated sales based only on pre-policy levels and trends (the counterfactual). We used segmented regression models to statistically estimate aggregate changes in levels and trends of monthly sales by volume from the pre-policy period to the post-policy period. Each model included a term to estimate the baseline trend, a binary indicator for all post-policy months to estimate the immediate level change in the outcome measure following the policy change, and a term indicating the number of months after policy implementation to estimate the change in trend (slope) during the post-policy period. The combined change in level and trend at a given month after the policy represented the full policy effect. To allow for the possibility of an anticipatory response to implementation of the policy, we considered a phase-in period of four months prior to the policy intervention in both countries and excluded these four data points from the time-series models [38-41]. We performed a sensitivity analysis by comparing results from interrupted times series models without a phase-in period. As the results were similar we display only the results from the models with a phase-in period. Further, we estimated absolute changes from the counterfactual one year after the policy intervention. We performed the analyses in SAS 9.3 and used a stepwise selection approach, which removed non-significant predictors (p > =0.2) from the model in order of least significance.

## Results

The antipsychotic market in Finland was dominated by three leading active substances: quetiapine (31%), clozapine (12%) and risperidone (12%), based on aggregated sales by volume from 2007 to 2011. In Portugal, risperidone (17%), quetiapine (17%) and amisulpride (12%) were the top three active substances sold in the class during that period.

**Figure 1:**
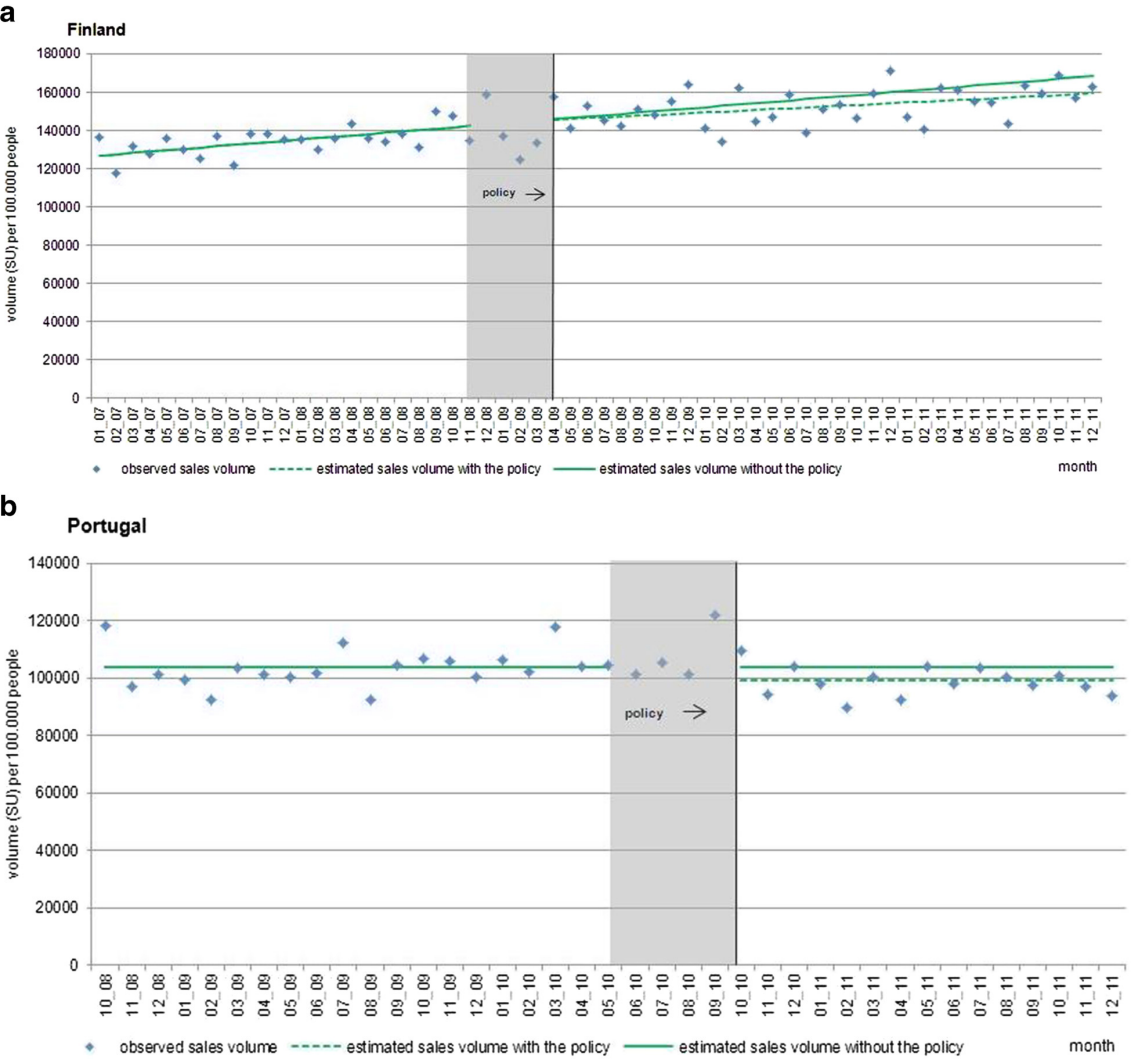
**Interrupted time series of the total retail antipsychotic markets in (a) Finland and (b) Portugal.** Observed values and interrupted time series estimates of the total retail antipsychotic market volume (standard unit per 100,000 persons per month) before and after the phase-in period of the policy interventions in Finland and Portugal. Data source: IMS MIDAS®, January 2007 and December 2011, IMS Health Incorporated. All Rights Reserved.

**Figure 2:**
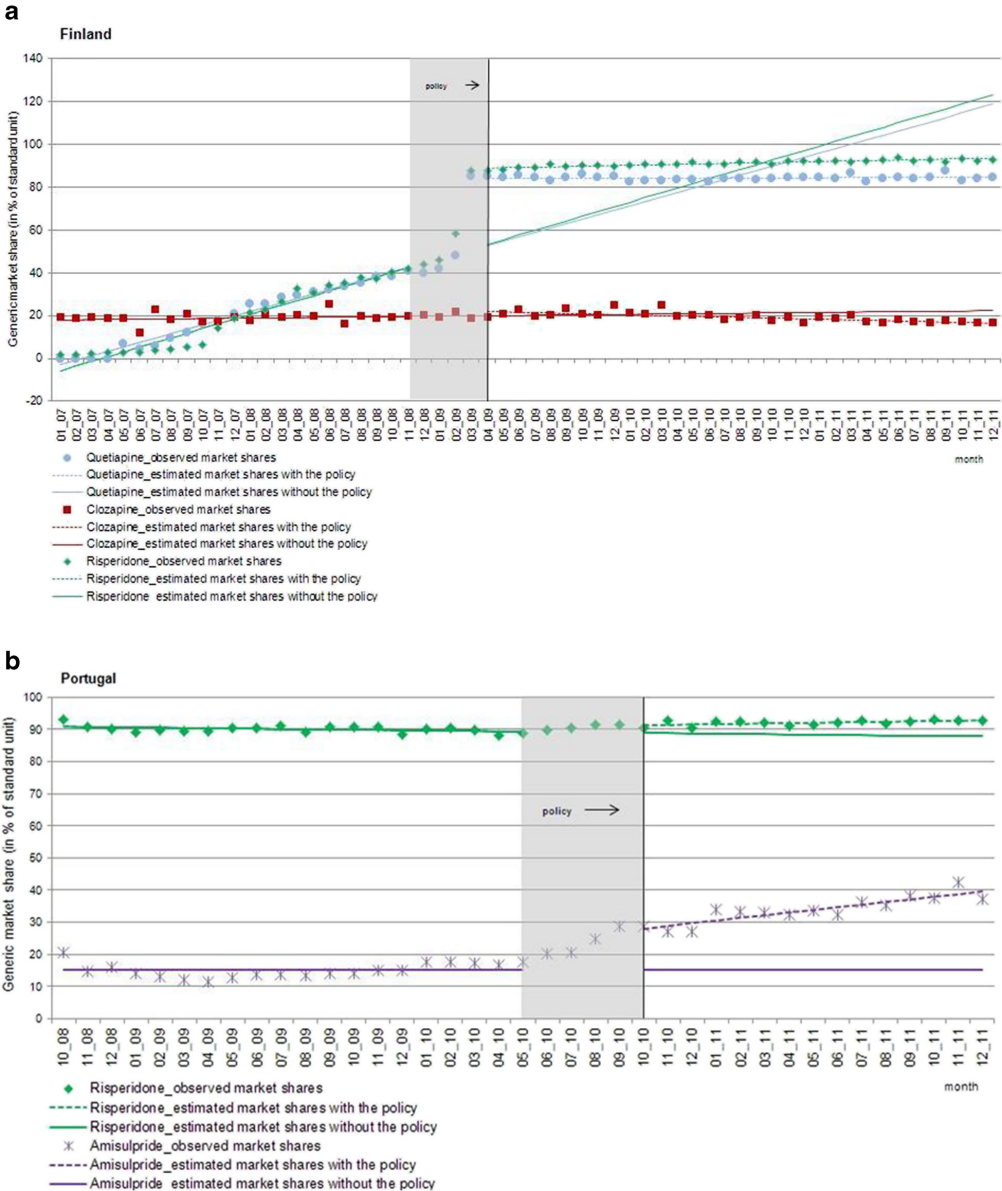
**Interrupted time series of retail generic market shares in (a) Finland and (b) Portugal.** Observed values and interrupted time series estimates of the retail generic market shares (percentage, standard units of generics per month) of the three top active substances in the antipsychotic market before and after the phase-in period of the policy interventions in Finland and Portugal. Data source: IMS MIDAS®, January 2007 and December 2011, IMS Health Incorporated. All Rights Reserved.

Figure 1(a,b) and Table 1 show the total monthly sales of antipsychotics in standard units per capita in both countries. In Finland, prior to the implementation of the reference price system, antipsychotic sales were increasing by 704 SU (95% CI: 519, 889) per 100,000 people per month; after the policy intervention, sales growth decreased by 273 SU (95% CI: -572, 26) per 100,000 people per month compared to pre-policy sales growth. There was no discontinuity in level of sales at the time of intervention. This resulted in an estimated, not statistically significant reduction of 3,550 SU (95% CI: -7,354, 254) per 100,000 people in actual sales compared to predicted sales (or around 2.3% of predicted sales) one year after the implementation of the reference price system. In Portugal, sales remained constant prior to the policies; however after the policy interventions, there was an estimated, statistically significant decrease in level of antipsychotic sales of 4,686 SU (95% CI: -8,913, -458) per 100,000 people (or 4.5% of predicted sales), which remained constant in the year after the policy.Figure 2(a,b) displays the time series of generic market shares as percentage of total standard units for the three leading antipsychotic substances in each country. We examined quetiapine, clozapine and risperidone in Finland and amisulpride and risperidone in Portugal; there were no generic quetiapine products on the market in Portugal at the time of the intervention.

**Table 1:** Estimates of baseline trend, level and trend changes, and absolute changes one year after the policy interventions for total antipsychotic sales and for generic market share of major active substances in Finland and Portugal

	**Variable**	**Monthly baseline trend pre-policy**	**Level change post-policy**	**Monthly trend change post-policy**	**Absolute estimated change one year post-policy**
	**(unit)**	**(95% CI)**	**(95% CI)**	**(95% CI)**	**(95% CI)**
**Finland**	Total sales growth per 100,000 population (SU)	704 (519, 889)	-	-273 (-572, 26)	-3550 (-7354, 254)
	Quetiapine generic market share (%)	2.07 (1.97, 2.17)	33.61 (31.61, 35.61)	-2.06 (-2.18, -1.95)	6.80 (3.92, 9.68)
	Risperidone generic market share (%)	2.19 (2.03, 2.34)	37.65 (34.60, 40.69)	-2.04 (-2.20, -1.88)	11.13 (6.79, 15.48)
	Clozapine generic market share (%)	0.07 (-0.02, 0.17)	2.22 (0.30, 4.14)	-0.25 (-0.36, -0.13)	-0.97 (-3.75, 1.81)
**Portugal**	Total sales growth per 100,000 population (SU)	-	-4686 (-8913, -458)	-	-4686 (-8758, -613)
	Risperidon generic market share (%)	-0.08 (-0.15, 0.01)	2.16 (0.76, 3.57)	0.19 (0.06, 0.31)	4.59 (2.67, 6.51)
	Amisulpride generic market share (%)	-	12.23 (9.61, 14.85)	0.82 (0.56, 1.09)	22.95 (21.01, 24.90)

CI = confidence interval, SU = standard unit.

Data source: IMS MIDAS®, January 2007 and December 2011, IMS Health Incorporated. All Rights Reserved.

In Finland, retail generic market share of all three antipsychotic substances was around 20% prior to the implementation of the reference price system with rapidly increasing generic market shares of risperidone (2.19% (95% CI: 2.03%, 2.34%) per month) and quetiapine (2.07% (95% CI: 1.97%, 2.17%) per month), while the increase in clozapine generic market share remained low (0.07% per month (95% CI: -0.02, 0.17)). After the implementation of the reference price system and the delisting of Seroquel, the generic market share of quetiapine increased abruptly by 33.61% (95% CI: 31.62%, 35.61%); accompanied by a decrease in market share growth of 2.06% (95% CI: -2.18%, -1.95%) per month, which resulted in an estimated increase of 6.80% (95% CI: 3.92%, 9.68%) generic market share one year after the policy implementation period. A similar abrupt shift to generics was seen for risperidone. After the introduction of the reference price system, there was an immediate increase of 37.65% (95% CI: 34.60%, 40.69%) in generic market share accompanied by a decrease in slope of 2.04% (95% CI: -2.20%, -1.88%) per month, which resulted in a generic market share that continued to increase slightly during the post-policy period. One year after implementation, we estimated an increase of 11.13% (95% CI: 6.79%, 15.48%) in generic market share due to the policies. Post-policy changes in generic market shares for clozapine were relatively minor compared to the other two active substances. After the introduction of the reference price system, there was a slight increase of 2.22% (95% CI: 0.30%, 4.14%) in generic market share accompanied by a slight decrease in slope of 0.25% (95% CI: 0.36%, -0.13%) per month, resulting in an estimated decrease of 0.97% (95% CI: -3.75%, -1.81%) generic market share one year post-policy.

In Portugal, the generic market prior to the multifaceted policy changes looked quite different from that in Finland. Generic market shares for amisulpride remained between 10% and 20% throughout the pre-policy period with no increasing trend, while the generic market share of risperidone was already around 90% prior to the policy. After implementation of the policies, there was an immediate increase in generic market share of 12.23% (95% CI: 9.61%, 14.85%) for amisulpride and an increase in market share trend of 0.82% (95%: 0.56%, 1.09%) per month, resulting in an estimated increase of 22.95% (95% CI: 21.01%, 24.90%) in total generic market share one year after the policy intervention. The market for risperidone had already been almost entirely generic prior to the reimbursement policy. After the policy, there was an estimated increase in generic market share of 2.16% (95% CI: 0.76%, 3.57%) and an increase in trend of 0.19% (95% CI: 0.06%, 0.31%) per month, resulting in an estimated increase of 4.59% (95% CI: 2.67%, 6.51%) in total generic market share one year after the policy mix. Details of the time series estimates and confidence intervals are summarized in Table 1.

## Discussion

Our analyses showed that both countries’ policy approaches were associated with an increased market share of generics as expected, but also with a likely unintended slight decrease in overall use of antipsychotic medicines, which may have been even more problematic against the background of increased need of antipsychotic medicines as a consequence of the economic recession. The decrease in overall use was expected in Portugal, but somewhat unexpected in Finland. In Finland two of the three leading active substances in the antipsychotic class (quetiapine and risperidone) experienced substantial increases in generic market share, but not the third substance (clozapine). In contrast, in Portugal the combination of policies which included statutory discounts granted to public payers, changes in reimbursement rates, and a generic campaign resulted in a major increase in generic market share only for one molecule (amisulpride); generic risperidone already dominated the market prior the policy interventions.

The two countries’ policy trajectories differed: The reference price system in Finland was prepared well in advance of implementation. In April 2003 Finland introduced mandatory generic substitution, requiring pharmacists to substitute higher-priced branded medicines with less-costly generic versions. The mandatory generic substitution at the time decreased average prices of substitutable medicines by more than 10% [42,43]. Among the key elements of guaranteeing a successful implementation of a generic policy is a transparent implementation process accompanied by early involvement of all stakeholders, such as doctors and pharmacists, as well as a detailed methodology and positive perceptions of patients towards generics [44,45]. System changes such as reference price systems are intended to facilitate changes in behaviors of patients and health providers by encouraging them to be more price-sensitive; in a reference price system patients have to pay out-of-pocket the difference between the reference price and the actual price, generating an incentive for patients to request a medicine that is priced at or below the reference price, usually a generic product. We demonstrated that Finland achieved the reference price policy goal of greater generic utilization. We also observed a reduction in utilization post-policy, which was gradual, not statistically significant and relatively smaller compared to Portugal.

In Portugal, we found that the mix of cost-containment measures that were ongoing before and after October 2010 led to a sudden, slight, statistically significant overall decrease of retail antipsychotic use – assuming a three tablet per day oral treatment, policy changes may have resulted in 6 to 97 fewer treatment days among 100,000 people per month in Portugal. We cannot disentangle which of the policies may have exerted most influence on utilization, or which population subgroups may have decreased utilization. At the end of 2010, Portugal was urgently seeking ways to cut public spending on medicines as the Portuguese economy had not recovered from the recession in 2009. Some of the policy measures shifted costs to patients by lowering reimbursement levels and requiring higher co-payments [46]. Shortly after the change in co-payment rates in 2010, public concerns in Portugal emerged that higher co-payments would have a negative impact on utilization of essential medicines including antipsychotic medicines [47]. Given that following the recession-induced policy package, patients in Portugal were no longer exempt from co-payments based on indication or other characteristics, it is reasonable to assume that sicker, poorer patients would be less likely to afford co-payment increased and more likely to decrease utilization.

In both countries, one of the most frequently sold active substances did not increase in generic market share (in Finland clozapine and in Portugal risperidone), which we attribute to different circumstances. Since clozapine use is associated with potentially life-threatening side effects, many countries have implemented strict prescription guidelines limiting clozapine prescriptions only to treatment-resistant schizophrenia patients [48]. Clinicians may also be reluctant to switch patients from clozapine to generic alternatives due to reports of worsening clinical status associated with generic substitution [49]. These clinical aspects may explain why clozapine use remained somewhat constant during the study period. Furthermore, the originator manufacturer of clozapine lowered its price to the reference price after the introduction of the reference price system, so there may not have been any financial incentive for patients to ask for a lower priced generic. Prescribing guidelines for the treatment of schizophrenia in Portugal recommended the use of risperidone and generic risperidone already had 90% of the market share prior to the policy interventions [50]. Due to the severity of the illness and strict prescribing guidelines the generic campaign in Portugal probably did not lead to increases in generic market share of this therapeutic group. Under these circumstances, the generic policies did not have an observable additional effect.

Adherence to therapies is especially challenging for patients on antipsychotic medicines. Increases in cost-sharing likely put additional pressure on vulnerable populations of low-income and chronically ill patients that may lead to lower utilization and worse health outcomes [51-53]. Soumerai et al. demonstrated that limits on coverage for the costs of outpatient prescription medicines can increase use of mental health services for acute exacerbations among low-income patients with chronic mental illnesses and result in increased costs to payers. He suggested that policy changes that pose substantial risks to vulnerable populations should undergo careful evaluation before their widespread adoption [54]. More research is needed on the potential unintended effects of reductions in reimbursement rates, as part of pharmaceutical sector responses to the recession, on utilization and long-term health outcomes.

IMS data represent country pharmaceutical markets consistently over time. They allow application of the strongest quasi-experimental research design for evaluating system-wide policy interventions. Nevertheless, the data pose some limitations. They do not allow us to determine the actual number of prescriptions issued or the actual amounts that third-party payers or patients pay for each medicine. We also did not have access to actual numbers of patients receiving antipsychotics nor could we determine the conditions for which antipsychotics were prescribed (including whether this was on- or off-label), or the characteristics of patients receiving them. Decreases in antipsychotic medicines use may have occurred among populations for whom the drugs were not indicated (constituting a desirable policy effect) or preferentially among poorer, sicker populations in need of the medicines (constituting an undesirable, inequitable policy effect). Other policies implemented at the same time could have confounded our analyses; to guard against this possibility, we have verified our policy data with policy makers from the PPRI network in each country. We recognize that we could not address the increased market share resulting from generics being on the market for a longer period of time, nor could we account for any economic incentives during this period that might have influenced prescribers’ behavior. We suggest that future research on the impacts of cost-containment policies on access to antipsychotics and other therapeutic classes should examine national prescribing data and national household survey data and use mixed methods to understand rationales for prescriber and patient behavior.

## Conclusions

Different policy approaches to contain pharmaceutical expenditures in the study countries had different intended – increased use of generics – and likely unintended – slightly decreased overall sales possibly consistent with decreased access to needed medicines – impacts. Especially the latter finding stresses the importance of examining the long-term effects of policy measures as increases in cost-sharing may have beneficial short-term impacts on public spending, but might also entail unintended long-term reductions in utilization, particularly for economically disadvantaged populations.

## Competing interest

All authors have completed the Unified Competing Interest form at http://www.icmje.org/coi_disclosure.pdf (available on request from the corresponding author) and declare: no support from any organisation for the submitted work; SV is employed by IMS, which provided the data free of charge. IMS is funded through sales of information and services to both industry and government, including all of the companies whose products are described in this study. AMT receive no direct funding or donations from private parties, including pharma industry. Research funding from public-private partnerships, e.g. IMI, TI Pharma (http://www.tipharma.nl<http://www.tipharma.nl>) has been accepted under the condition that no company-specific product or company related study is conducted. They have received unrestricted research funding from public sources, i.e. the Netherlands Organisation for Health Research and Development (ZonMW), the Dutch Health Care Insurance Board (CVZ), the EU 7th Framework Program (FP7), the Dutch Medicines Evaluation Board (MEB), and the Dutch Ministry of Health. The results present in this study are the personal views of the authors.

## Authors’ contributions

CL has made substantial contributions to the conception and design, carried out the analysis and drafted the manuscript. FZ performed the statistical analysis. AKMT and AKW contributed to the formulation of the research question and the study design were involved in the analysis and helped draft the manuscript. SV provided the data and helped to understand and interpret the data correctly. SV and DRD made substantial contributions to the conception and design and critically revised the manuscript. All authors read and approved the final manuscript.

## Authors’ information

This research was conducted while CL was a Visiting Scholar at the Department of Population Medicine, Harvard Medical School and Harvard Pilgrim Health Care Institute.

## Supplementary Material

Additional file 1 List of products included in the study. Click here for file
