# Supplementary material for: Impact of pharmaceutical policy interventions on utilization of antipsychotic medicines in Finland and Portugal in times of economic recession: interrupted time series analyses
Source: Int J Equity Health. 2014 Jul 25;13:53. doi: 10.1186/1475-9276-13-53 (PMC4126811; doi:10.1186/1475-9276-13-53)
Supplement: Additional file 1 — List of products included in the study. [file 1475-9276-13-53-S1.docx]

Annex: Table List of products included in the study

| **Country** | **Active substance** | **Product** | **Generic / Original** |
| --- | --- | --- | --- |
| Finland | QUETIAPINE | KETIPINOR | Generic |
|  |  | QUETIAPIN RATIOPHA | Generic |
|  |  | QUETIAPIN MYLAN | Generic |
|  |  | QUETIAPIN HEXAL | Generic |
|  |  | QUETIAPIN ACTAVIS | Generic |
|  |  | SEROQUEL PROLONG | Original |
|  |  | SEROQUEL | Original |
|  | RISPERIDONE | RISPERIDON ORION | Generic |
|  |  | RISPERIDON RATIOPH | Generic |
|  |  | RISPERIDON SANDOZ | Generic |
|  |  | RISPERIDON MYLAN | Generic |
|  |  | RISPERIDONE STADA | Generic |
|  |  | RISPERIDON TEVA | Generic |
|  |  | RISPERIDON ORIFARM | Generic |
|  |  | RISPERIDON ACTAVIS | Generic |
|  |  | RISPERIDON KRKA | Generic |
|  |  | RISPAZIN | Generic |
|  |  | RISPERIDON HEXAL | Generic |
|  |  | RISPERDAL | Original |
|  |  | RISPERDAL CONSTA | Original |
|  | OLANZAPINE | OLANZAPIN RATIOPHA | Generic |
|  |  | OLANZAPIN LILLY | Generic |
|  |  | OLANZAPINE GLENMAR | Generic |
|  |  | OLANZAPIN ORIFARM | Generic |
|  |  | OLANZAPIN ACTAVIS | Generic |
|  |  | OLANZAPIN SANDOZ | Generic |
|  |  | OLANZAPINE MYLAN | Generic |
|  |  | OLANZAPINE NYZOL | Generic |
|  |  | OLANZAPIN TEVA | Generic |
|  |  | SOLAZIN | Generic |
|  |  | ZALASTA | Generic |
|  |  | OLANZAPIN BLUEFISH | Generic |
|  |  | ZYPREXA | Original |
|  |  | ZYPADHERA | Original |
|  |  | ZYPREXA PAR | Original |
|  | LEVOMEPROMAZINE | LEVOZIN | Generic |
|  |  | NOZINAN | Original |
|  | CLOZAPINE | FROIDIR | Generic |
|  |  | CLOZAPINE ACTAVIS | Generic |
|  |  | CLOZAPIN HEXAL | Generic |
|  |  | LEPONEX | Original |
|  |  | LEPONEX ORI | Original |
|  |  | LEPONEX PAR | Original |
|  | CHLORPROMAZINE | KLORPROMAN | Generic |
|  |  | TRUXAL | Original |
|  | MELPERONE | MELPAX | Generic |
|  | PERPHENAZINE | PERATSIN | Original |
|  | ARIPIPRAZOLE | ABILIFY | Original |
|  | HALOPERIDOL | SERENASE J-C | Original |
|  | PROCHLORPERAZINE | STEMETIL | Original |
|  | FLUPENTIXOL | FLUANXOL | Original |
|  | SULPIRIDE | SUPRIUM | Original |
|  | ZUCLOPENTHIXOL | CISORDINOL | Original |
|  | PERICIAZINE | NEULACTIL | Original |
|  | ZIPRASIDONE | ZELDOX | Original |
|  | SERTINDOLE | SERDOLECT | Original |
|  | SYCREST | ASENAPINE | Original |
|  | FLUPHENAZINE | SIQUALONE | Original |
|  | PALIPERIDONE PALMITATE | XEPLION | Original |
|  | MELPERONE | BURONIL | Original |
|  | DIXYRAZINE | ESUCOS | Original |
| Portugal | QUETIAPINE | SEROQUEL | Original |
|  |  | ALZEN | Original |
|  |  | KVENTIAX MG | Generic |
|  |  | QUETIAPINA MG WY | Generic |
|  |  | QUETIAPINA MG GES | Generic |
|  |  | QUETIAP MG QUALIGE | Generic |
|  |  | QUETIAPINA MG ALT | Generic |
|  | MELPERONE | BUNIL | Original |
|  | OLANZAPINE | ZYPREXA | Original |
|  | AMISULPRIDE | SOCIAN | Original |
|  |  | AMISSULPRID ACT | Generic |
|  |  | AMISSULPRID GES | Generic |
|  |  | AMISSULPRID SDZ | Generic |
|  |  | AMISSULPRID MYN | Generic |
|  |  | AMISSULPRID WY | Generic |
|  |  | AMITREX | Generic |
|  | CYAMEMAZINE | TERCIAN | Original |
|  | HALOPERIDOL | HALDOL | Original |
|  | SULPIRIDE | DOGMATIL | Original |
|  | TIAPRIDE | TIAPRIDAL | Original |
|  |  | TIAPRIDA GES | Generic |
|  | LEVOMEPROMAZINE | NOZINAN | Original |
|  | ARIPIPRAZOLE | ABILIFY | Original |
|  | CHLORPROMAZINE | LARGACTIL | Original |
|  | CLOZAPINE | LEPONEX | Original |
|  |  | CLOZAPINA GES | Generic |
|  |  | CLOZAPINA SDZ | Generic |
|  | RISPERIDONE | RISPERDAL | Original |
|  |  | RISPER RAT | Generic |
|  |  | RISPER GES | Generic |
|  |  | RISPER TLF | Generic |
|  |  | RISPER WY | Generic |
|  |  | PERDIN | Generic |
|  |  | RISPER ALT | Generic |
|  |  | RISPER DHIRA | Generic |
|  |  | RISPER MYN | Generic |
|  |  | RISPER MPH | Generic |
|  |  | RISPER SDZ | Generic |
|  |  | RISPER CCL | Generic |
|  |  | RISPER BLU | Generic |
|  |  | RISPER TEV | Generic |
|  |  | RISPER GPO | Generic |
|  |  | RISPER AOB | Generic |
|  |  | RISPER GMD | Generic |
|  |  | RISPER BAS | Generic |
|  |  | RISPER KK. | Generic |
|  |  | RISPER ACT | Generic |
|  |  | RISPER LGC | Generic |
|  |  | RISPER BAL | Generic |
|  |  | RISPER GNC | Generic |
|  |  | RISPER PHM | Generic |
|  |  | RISPER AZE | Generic |
|  |  | RISPER CIN | Generic |
|  |  | LOTIN | Generic |
|  |  | ZORIDAL | Generic |
|  |  | RISPER VIDA | Generic |
|  |  | RISPER P.D | Generic |
|  |  | RISPER A&P | Generic |
|  |  | RISPER ARW | Generic |
|  |  | RISPER RBY | Generic |
|  |  | RISPER ZVA | Generic |
|  |  | AZIROSAMA | Generic |
|  | PALIPERIDONE | INVEGA | Original |
|  | ZIPRASIDONE | ZELDOX | Original |
|  | ZOTEPINE | ZOLEPTIL | Original |
|  | PIMOZIDE | ORAP | Original |
|  | FLUPENTIXOL | FLUANXOL | Original |
|  | ZUCLOPENTHIXOL | CISORDINOL | Original |
|  | FLUPHENAZINE | ANATENSOL DECANOAT | Original |
|  | SERTINDOLE | SERDOLECT | Original |
|  | SULPIRIDE | LISOPIRIDE | Original |
|  | THIORIDAZINE | MELLERIL | Original |
|  | HALOPERIDOL | SERENELFI | Original |
|  |  | HALOPERIDOL RAT | Generic |
